# Bilateral Bochdalek Hernias Associated with Arnold-Chiari I Malformation

**DOI:** 10.1155/2020/1931879

**Published:** 2020-01-22

**Authors:** Julian Fazi, Visad Patel, Cara Bryan

**Affiliations:** West Virginia University Department of Radiology, Morgantown WV 26505, USA

## Abstract

A Bochdalek hernia is a posterolateral diaphragmatic defect that is either congenital or acquired. The contents of the hernia range from fat to intra-abdominal organs. They are primarily pathologies of neonates and most commonly occur unilaterally. These hernias have been described in isolation and as one part of a group of malformations. There have been reports of Bochdalek hernias in association with myelomeningocele and other neural tube defects. We present a unique case of bilateral Bochdalek hernias in a 35-year-old female with an Arnold-Chiari I malformation.

## 1. Introduction

The most common type of congenital diaphragmatic hernia, which is defined as the failure of the pleuroperitoneal canal to form properly, is the Bochdalek hernia [1]. A Bochdalek hernia is a diaphragmatic hernia that arises due to failure of the posterolateral foramina to close properly (congenital) or failure to remain closed (acquired) [2]. Fat, omentum, and less commonly intra-abdominal organs protrude into the thorax; however, thoracic organs have not been reported to herniate into the abdomen [2–4]. This is primarily a pathology of neonates, but it can affect children and adults, although much less frequently [1, 2, 5]. Asymptomatic Bochdalek hernias are reported in 0.17–12.7% of subjects, but they infrequently occur bilaterally [2, 6]. Furthermore, there have been few reports of Bochdalek hernias associated with myelomeningocele and other neural tube defects [7–9], but, to our knowledge, there have been no reports associated with an Arnold-Chiari I malformation.

## 2. Case Presentation

**Figure 1:**
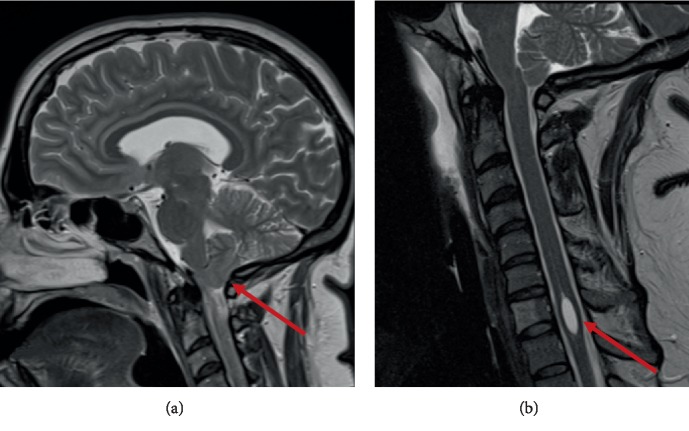
T2-weighted MRI demonstrating Arnold-Chiari I malformation. (a) shows a 7 mm downward displacement of the right cerebellar tonsil (red arrow). (b) shows a syrinx centered about C7-T1 measuring 17 mm and 5.6 mm in the largest longitudinal and axial dimension (red arrow). A benign hemangioma of C7 anterior vertebral body is also identified.

A 35-year-old female with a past medical history of severe obesity, gastroesophageal reflux disease (GERD), scoliosis, anxiety, depression, bipolar disorder, and migraines developed blurry vision, pain, numbness, and weakness in her extremities over a six-month time period that required her to use a walker due to frequent falls. Her neurologist ordered a magnetic resonance imaging (MRI) scan with and without contrast of the cervical, thoracic, and lumbosacral spines that showed mild downward displacement of the cerebellar tonsils (7 mm) without evidence of hydrocephalus (Figure 1). These findings are consistent with an Arnold-Chiari I malformation which is defined as a 5 mm or greater descent of the cerebellar tonsils through the foramen magnum diagnosed via autopsy or radiography [10]. The findings were confirmed by a cine MRI. The MRI also showed a cord syrinx measuring 17 mm in longitudinal dimension with the greatest axial diameter of 5.6 mm at C7-T1 (Figure 1).

**Figure 2:**
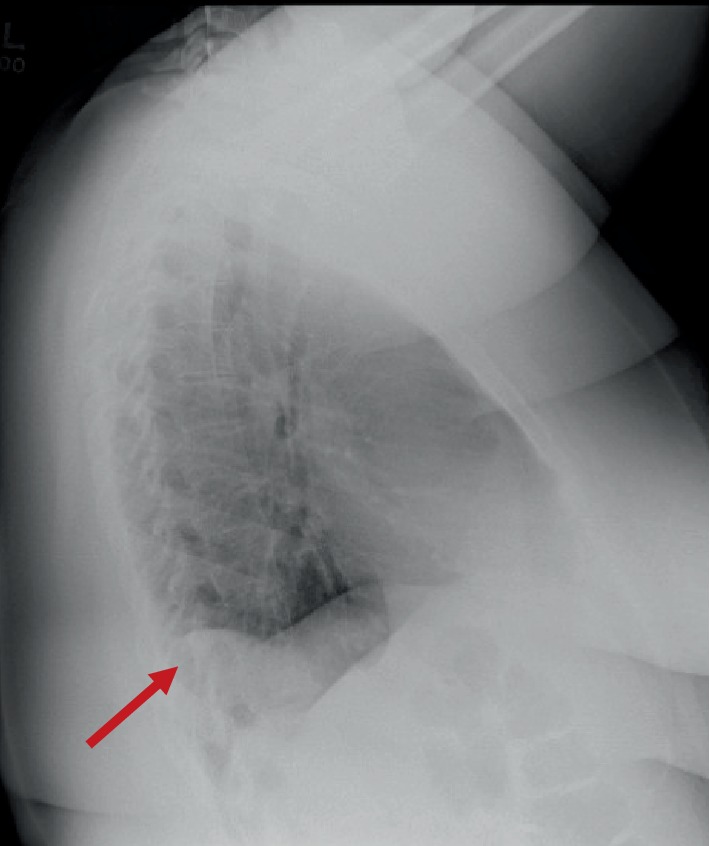
Lateral chest radiograph showing a mass density in the right posterior costophrenic angle with diaphragmatic contour irregularity representing the right side of the bilateral Bochdalek hernia.

A chest radiograph was performed during the same admission due to peripheral edema in the setting of arrhythmias. The lateral chest radiograph demonstrated a mass density in the right posterior costophrenic angle suggestive of a possible Bochdalek hernia (Figure 2). The patient underwent a suboccipital craniectomy, C1 laminectomy, and expansion duraplasty due to blurry vision, pain, numbness, and weakness in her extremities. There were no immediate complications. She followed up in a clinic 11 days postoperatively and reported numbness in her hands, tremors in her hands and feet, headaches, and constipation.

Six weeks postoperatively, she presented to the emergency department (ED) complaining of drainage from her suboccipital craniectomy site without fever, chills, or other signs of infection. Computed tomography (CT) scan demonstrated a large pseudomeningocele in the decompression bed, so a lumbar drain was placed at the L3-L4 level using interventional radiology (IR) guidance. The lumbar drain did not produce a significant amount of cerebrospinal fluid (CSF), and the pseudomeningocele persisted, so it was determined that it was necessary to place a ventriculoperitoneal (VP) shunt. After placement of the VP shunt, the drainage decreased, and the patient was determined safe for discharge since she was tolerating a diet, physical therapy, and ambulation. CSF and blood cultures were also negative. The patient returned multiple times after the immediate postoperative period with persistent headaches, numbness, and weakness despite her neurosurgical intervention.

**Figure 3:**
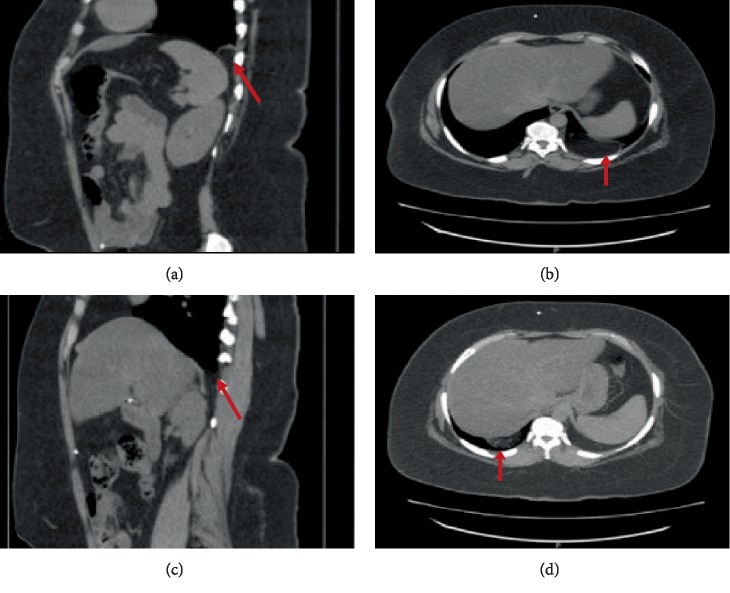
Computed Tomography (CT) scan of the abdomen showing a 1.6 cm left-sided Bochdalek Hernia in a sagittal (a) and axial (b) plane containing fat (red arrow) and a 1.5 cm right-sided Bochdalek Hernia in a sagittal (c) and axial (d) plane containing only fat (red arrow).

Six months later, the patient was evaluated by thoracic surgery for a potential Bochdalek hernia as seen on lateral chest radiograph in the setting of GERD. CT of the chest and abdomen demonstrated bilateral posterolateral diaphragmatic defects measuring 1.6 cm on the left and 1.5 cm on the right consistent with bilateral Bochdalek hernias (Figure 3). Since the hernias were small, contained only intraperitoneal fat, and no hiatal hernia was present, it was determined that surgery would most likely not help her GERD symptoms and thus was not warranted.

## 3. Discussion

Congenital diaphragmatic hernias occur in approximately 1 in 2,500 live births, and 70-75% of these are posterolateral (Bochdalek) defects [11]. The diaphragm forms between the fourth and twelfth week of gestation and arises from four components: the septum transversum, pleuroperitoneal membranes, dorsal mesentery of the esophagus, and body wall musculature [12, 13]. Ultimately, the diaphragm is the product of three muscle groups: the pars costalis, pars sternalis, and pars lumbaris [12]. Gaps between these muscles or failure of these muscles to join properly can lead to various hernias [12]. Bochdalek hernias are the most common type of congenital diaphragmatic hernias and are a product of aberrant joining of the pars costalis and the pars lumbaris resulting in a posterolateral diaphragmatic defect [2, 12]. These hernias are typically pathologies of neonates with most diagnoses occurring before or shortly after birth and only 5-25% after eight weeks of age [1, 12, 14]. Nonetheless, they have been identified in children and older adults [2]. Neonates and young infants have predominantly respiratory symptoms from maldevelopment and compression and of the lungs [5, 11]. Older children are more likely to present with gastrointestinal symptoms [1, 15], and adults are frequently diagnosed by imaging performed for other reasons as seen with our patient [2, 15]. When seen in an older population, the question arises whether or not the defect was congenital or acquired. Examples of acquired causes include blunt or penetrating trauma, labor, and physical exertion [16]. However, some argue that acquired defects may be congenital defects that were too small to be detected prior to the insult [12]. As for our patient, it is possible that her diaphragmatic defects could have been small at birth and further accentuated by her large body habitus.

Detection of Bochdalek hernias through imaging performed for other reasons is not uncommon. Imaging characteristics consist of an interrupted diaphragm with hernia contents that range from intra-abdominal and retroperitoneal fat to intra-abdominal and retroperitoneal organs [12]. There was a high suspicion on the chest radiograph in the case presented, but a CT scan was needed to both confirm the diagnosis and determine the contents of the hernia. Multidetector row CT (MDCT) with multiplanar and three-dimensional imaging helps make the diagnosis as opposed to using conventional CT alone [17]. The use of this technology has allowed for increased diagnosis of the hernias in asymptomatic individuals. For example, Kinoshita et al. report a prevalence of 12.7% with the use of MDCT [6] compared with Mullins et al. who used conventional CT and found a 0.17% prevalence [2]. In the case of neonates, ultrasound has been shown to be a useful tool in determining the correct surgical approach (open versus laparoscopic) if surgery is deemed necessary [18].

Approximately 50-60% of congenital diaphragmatic hernias are isolated defects, and 40-50% are associated with either a syndrome or other congenital defects not designated to a particular syndrome [19]. Commonly affected organ systems include the pulmonary (pulmonary hypoplasia and pulmonary arterial hypertension), gastrointestinal, and neurological systems [19]. There have been multiple reports of congenital diaphragmatic hernias associated with myelomeningoceles [7], and there has been a report of congenital diaphragmatic hernia associated with an Arnold-Chiari II malformation [4]. To our knowledge, this is the first report associated with an Arnold-Chiari I malformation. Despite the fact that an embryological association has not been found between the Arnold-Chiari I malformation and bilateral Bochdalek hernia, our experience suggests that if a patient presents with a known Arnold-Chiari I malformation and symptoms suggestive of a Bochdalek hernia, investigation of the diaphragmatic hernia may be warranted.

## 4. Conclusion

Bochdalek hernias are posterolateral diaphragmatic hernias and are the most common type of congenital diaphragmatic hernia. They can either be congenital and arise due to failure of fusion of the diaphragm in early development or be acquired due to various mechanisms. They are most commonly diagnosed in the perinatal period, but they can be diagnosed into late adulthood depending on the severity of the defect and symptoms. These hernias can either be an isolated defect or one part of a host of malformations. The case presented is the first reported bilateral Bochdalek hernia associated with an Arnold-Chiari I malformation.
